# Visceral Leishmaniasis in a UK Toddler following a Short Trip to a Popular Holiday Destination in Spain

**DOI:** 10.1155/2014/537052

**Published:** 2014-08-10

**Authors:** Neda Minakaran, Talha Soorma, Shamez N. Ladhani

**Affiliations:** ^1^The Whittington Hospital NHS Trust, Magdala Avenue, London N19 5NF, UK; ^2^Barts and The London School of Medicine and Dentistry, Turner Street, London E1 2AD, UK; ^3^St. George's Hospital NHS Trust, Blackshaw Road, London SW17 0QT, UK

## Abstract

We herein present the case of a 15-month-old with visceral leishmaniasis diagnosed in the UK following a short trip to a popular holiday destination in Spain. Four months after the initial symptoms, the diagnosis was made incidentally on microscopy of a bone marrow biopsy taken for suspected haematological malignancy after the child developed hepatosplenomegaly, pancytopaenia, and *Klebsiella pneumoniae* septicaemia.

## 1. Introduction

Leishmaniasis is a zoonotic parasitic disease with a reservoir of dogs, wild rodents, and other animals in endemic areas. It is transmitted by the female phlebotomine sandfly and humans are an accidental host [1]. Currently, there are an estimated 12 million people affected worldwide [2]. Clinically, leishmaniasis can be subdivided into cutaneous, visceral, and mucocutaneous types, each being transmitted by different species and subspecies of the sandfly, producing a diverse spectrum of symptoms.

Visceral leishmaniasis (kala-azar, meaning “black fever”; VL) is a progressive systemic disease which, if left untreated, has an almost 100% fatality rate within two years [1]. Common presenting features include irregular bouts of fever, substantial weight loss, hepatosplenomegaly, and anaemia. An estimated 400,000 new cases of VL [2] and around 50,000 deaths occur worldwide each year [3], with more than 90% of global VL cases occurring in six countries: India, Bangladesh, Sudan, South Sudan, Ethiopia, and Brazil [2]. Leishmaniasis does not occur in the UK and all cases are presumed to have been acquired abroad [2]. For all types of leishmaniasis diagnosed in the UK during 2006 and 2007, the top four countries of travel were Belize (11 in 2006, 3 in 2007), Iraq (7 in 2006, 1 in 2007), Afghanistan (3 in both 2006 and 2007), and Spain (1 in 2006 and 4 in 2007) [4]. Although Mediterranean countries contribute to only a small proportion of VL cases globally, they are one of the major sources of imported VL diagnosed in the UK because of high rates of tourism to these countries by British citizens [5]. Here, we report the youngest UK case of VL in a 15-month-old immunocompetent child who acquired the disease during a very short stay in the popular holiday destination of Alicante, Spain.

## 2. Case Report

A 15-month-old Caucasian girl presented to her local hospital with a five-month history of intermittent fevers and weight loss. During this period, she had been seen by her general practitioner several times and referred to the emergency department on two separate occasions, where she had been diagnosed with a viral upper respiratory tract infection and a possible urinary tract infection. She was born at term and was up-to-date with immunisations, gaining weight, and developing appropriately for her age. Her past medical history was unremarkable with no prior admissions to hospital. She was an only child and there was no relevant family history. Her only travel history was a one-week holiday to Lliber in the Jalon Valley of Alicante, Spain, seven months previously.

On examination, she was pale and febrile at 41°C, with hepatomegaly of 2 cm and splenomegaly of 4 cm below the costal margins, respectively. Haematological investigations showed pancytopaenia, with haemoglobin concentrations of 7.6 g/dL, white cell count 4.4 × 10^9^/L, neutrophils 0.5 × 10^9^/L, and platelets 65 × 10^9^/L. Her CRP was raised at 69 mg/dL. An abdominal ultrasound scan confirmed hepatosplenomegaly but did not identify any intra-abdominal lymphadenopathy. Although the peripheral blood film did not show any blast cells, a provisional diagnosis of acute lymphoblastic leukaemia was made and she was commenced on piptazobactam and gentamicin whilst awaiting transfer to a specialist oncology centre.

Over the next few days, her haemoglobin and platelet levels dropped further, requiring multiple transfusions. She had two bone marrow aspirates, neither of which showed evidence of acute leukaemia on immunophenotyping. Repeated blood, stool, urine, and throat bacterial cultures as well as viral serological tests done at presentation and during admission remained negative. After five days of antibiotic therapy, she was started on liposomal amphotericin B at a dose of 1 mg/kg/day but this was stopped within 48 hours because her condition improved and she became afebrile. Her antibiotics were stopped three days later and she was discharged home whilst awaiting the results of her bone marrow biopsy.

Two days later, she was readmitted with fever, and piptazobactam and gentamicin antibiotics were resumed. On this occasion, her blood cultures grew* Klebsiella pneumoniae*. At the same time, microscopy of her bone marrow biopsy was reported positive for leishmaniasis, which was subsequently confirmed by serology. She was, therefore, commenced on liposomal amphotericin B at 3 mg/kg/day. Her fever settled after 48 hours and, on day five, her neutrophil count increased to 1.4 × 10^9^/L. She completed seven days of treatment and was discharged from hospital, to return for a further dose of liposomal amphotericin B on days 10 and 20. At three-month follow-up, she remained well with resolution of her hepatosplenomegaly, a normal blood count, and no further episodes of fever.

## 3. Discussion

This case is the youngest child to be diagnosed with VL in the UK, following a very short holiday to a popular holiday destination resort in mainland Spain. The delay of several months before the diagnosis was made highlights the difficulties in diagnosis of such a rare imported disease, particularly in a child, as the nonspecific presenting features can be attributed to a number of common self-limiting childhood illnesses.

VL is caused by the* Leishmania donovani *complex,* L. donovani* sensu stricto in East Africa and the Indian subcontinent, and* L. infantum* in Europe, North America, and Latin America [1]. VL is usually considered a disease of the tropics. However, the disease is also endemic in southern European countries, many of which are popular holiday destinations for British tourists. Contrary to their name, the sandflies that transmit the parasite are usually found in forest areas, caves, and burrows of small rodents. They predominantly bite between dusk and dawn and usually stay close to the ground.

Leishmaniasis does not occur naturally in the UK and data on VL in England are sparse because VL notification is not mandatory. Voluntary reporting of cases to Public Health England, however, has increased markedly in recent years, from fewer than 20 annual cases of leishmaniasis reported before 2001 to >50 during 2005–2008 [6]. VL accounts for a small proportion of the total cases, with 4, 1, 6, 6, and 6 annual cases reported during 2002–2006, respectively, although there was a rise to 14 in 2007 and 17 in 2008. Of these, only one was a child, a fourteen-year-old boy who had acquired the infection in Pakistan [7].

In England, most cases are likely to be referred to one of the two national centres for tropical medicine in London and Liverpool. In London, the Hospital of Tropical Diseases identified 39 VL cases managed at their hospital during 1985–2004, representing ~83% of all UK cases, and most cases (77%) were imported from Mediterranean countries, especially Spain (43%) [5]. Interestingly, another report by the same centre detailing cases of imported cutaneous leishmaniasis diagnosed during 1998–2009 also found that the majority (71%) of the 223 patients were tourists to the Mediterranean region [8].

Clinically, a significant proportion of cases are asymptomatic. Those causing clinical symptoms, however, can follow an acute, subacute, or chronic course with an incubation period ranging from weeks to months. The clinical features of VL include fever, fatigue, pallor, loss of appetite, weight loss, generalised lymphadenopathy, and hepatosplenomegaly. Laboratory investigations may identify hypogammaglobulinaemia and/or pancytopaenia due to parasitic invasion of the bone marrow and the reticuloendothelial system. Untreated, patients usually die from anaemia, severe invasive bacterial infections secondary to neutropenia, massive bleeding due to low platelet counts, and/or malnutrition.

In our case, an imported infection was not considered in the differential diagnosis because of the short duration of travel to a popular holiday destination resort.

Even after investigations revealed pancytopaenia, a childhood haematological malignancy was considered the most likely diagnosis and VL was diagnosed incidentally on microscopy of a bone marrow aspirate, which identified amastigotes in clinical specimens.

The diagnostic sensitivity for light microscopy of splenic, bone marrow, and lymph node aspirate smears is >95%, 55–97%, and 60%, respectively [1]. In endemic areas, the skill and resources to safely perform these procedures and interpret their results may not be available, and so “field tests” play an important role in diagnosis. These include serological tests such as the direct agglutination test (sensitivity 94.8%, specificity 85.9%) [9] and the rapid rK39 dipstick test (sensitivity 91.9%, specificity 92.4%) [10]. However, due to the number of people in endemic communities with asymptomatic infection, these tests can never be used as stand-alone diagnostic tests in persons without clinical signs. Similarly, species-specific PCR, whilst highly sensitive, is usually only available in specialist centres and, again, can have low specificity in endemic populations due to high numbers of asymptomatic carriers. In one study,* Leishmania* DNA was amplified in 38.2% of blood samples from endemic non-VL patients with fever and splenomegaly due to other aetiology [11].

Prior to 1995, patients with VL in the UK were treated with multiple drugs such as sodium stibogluconate, paromomycin, meglumine antimoniate, and pentamidine [5]. Since then, liposomal amphotericin B has become the drug of choice, being used in >80% of cases globally, although its high costs mean that availability is low in developing countries, which often have the highest burden of disease [5]. Successful treatment of VL should also include aggressive management of anaemia, thrombocytopenia, and any infections secondary to neutropenia.

In summary, our case demonstrates the importance of taking a detailed and accurate travel history, irrespective of the duration of travel and regardless of the traveller's age. Our case also highlights the importance of considering leishmaniasis in the differential diagnosis of imported infection among travellers to Mediterranean countries, especially in the presence of hepatosplenomegaly and/or pancytopaenia.

With increasing ease of travel, the number of British tourists travelling abroad for vacations each year continues to rise. A formal notification system for rare imported infections such as VL does not yet exist in the UK but, if established, would be of great benefit for monitoring epidemiological trends and health planning. It is also important that clinicians seek specialist advice and consider early referral when managing children with suspected imported infections.

## Abbreviations

VL: Visceral Leishmaniasis
UK: United Kingdom
HIV: Human immunodeficiency virus
AIDS: Acquired immunodeficiency syndrome
WHO: World Health Organisation.
